# Preparation and Characterization of a Novel Morphosis of Dextran and Its Derivatization with Polyethyleneimine

**DOI:** 10.3390/molecules28207210

**Published:** 2023-10-21

**Authors:** Zhiming Jiang, Kaifeng Sun, Hao Wu, Weiliang Dong, Jiangfeng Ma, Min Jiang

**Affiliations:** State Key Laboratory of Materials-Oriented Chemical Engineering, College of Biotechnology and Pharmaceutical Engineering, Nanjing Tech University, Nanjing 211800, China

**Keywords:** dextran, hydrogen bonding, polymorph formation, support of enzyme immobilization

## Abstract

Dextran, a variant of α-glucan with a significant proportion of α-(1,6) bonds, exhibits remarkable solubility in water. Nonetheless, the precipitation of dextran has been observed in injection vials during storage. The present study aimed to establish a technique for generating insoluble dextran and analyze its structural properties. Additionally, the potential for positively ionizing IS-dextran with polyethyleneimine was explored, with the ultimate objective of utilizing IS-dextran-PEI as a promising support for enzyme immobilization. As a result, IS-dextran was obtained by the process of slow evaporation with an average molecular weight of 6555 Da and a yield exceeding 60%. The calculated crystallinity of IS-dextran, which reaches 93.62%, is indicative of its irregular and dense structure, thereby accounting for its water insolubility. Furthermore, positive charge modification of IS-dextran, coupled with the incorporation of epichlorohydrin, resulted in all zeta potentials of IS-dextran-PEIs exceeding 30 mV, making it a promising supporting factor for enzyme immobilization.

## 1. Introduction

The regulation of specific polymorph formation is a matter of significant importance, as distinct polymorphs exhibit varying physical properties, including water solubility, stability, and bio-availability [1]. The preparative technique can result in the formation of different polymorphs from identical starting components. For example, glycine, the smallest amino acid, has been utilized as a model substance for polymorphism, with α-glycine and β-glycine being the primary products of slow evaporation and rapid precipitation, respectively [2]. The results of crystallization can vary depending on the rate of evaporation, with differences observed between slow and rapid evaporation methods, such as oven drying and spray drying. Maidur et al. have documented the successful preparation of optically transparent single crystals of two pyridine-based anthracene chalcones using the slow evaporation solution growth technique [3]. Similarly, Hetmanczyk et al. have reported the formation of two polymorphic forms of hydroxy acetophenone through slow and fast evaporation of polar and non-polar solutions, respectively [4].

Dextran is an α-glucan variant characterized by a continuous main chain of α-(1,6) glycosidic bonds and branch linkage through α-(1,2), α-(1,3), and α-(1,4) glycosidic bonds [5]. The high proportion of α-(1,6) bonds in dextran confers high solubility in water, whereas an increased proportion of α-(1,3) bonds reduces solubility. Mutan, which comprises a high ratio of α-(1,3)-linked glycosidic bonds, is nearly insoluble in water due to the formation of strong hydrogen bonds [6]. However, dextran precipitation has been observed in injection vials during storage, and further investigation is necessary to elucidate the underlying insolubilization mechanism. Hirata and colleagues discovered that the presence of boron resulted in the aggregation of dextran, as determined by dynamic light-scattering measurements and X-ray-scattering techniques. Furthermore, the precipitation of dextran was found to be dependent on its molecular weight [7,8]. Dextrans lacking boron and possessing higher molecular weights (dextran 40,000 Da and 220,000 Da) exhibited resistance to precipitation, while concentrated aqueous solutions of low-molecular-weight dextran (dextran 6000 Da) were observed to form microspheres or gels [9]. Dextran has favorable physicochemical properties, physiological compatibility, and minimal cytotoxicity toward cells, thus presenting extensive potential for various applications. Hydrophobically modified dextrans with surface-active characteristics have been employed as effective emulsion stabilizers, replacing conventional emulsifiers [10]. Additionally, self-assembled microspheres formed from insoluble precipitates in dextran solutions have shown the ability to encapsulate and subsequently release proteins, indicating promising prospects as drug carriers [11]. Furthermore, the utilization of dextran hydrogels as drug carriers for sustained and controlled release was facilitated through the physical cross-linking of aqueous dextran solutions [9]. Additionally, the construction of an interfacial coating interlayer on polysulfone (PSF) ultrafiltration (UF) substrate was achieved by crosslinking polyethyleneimine (PEI) and dextran nanoparticles (DNPs) using glutaraldehyde (GA). This innovative approach demonstrated significant promise in the domains of rapid desalination and wastewater recovery [12].

Currently, the immobilization of enzymes is a viable approach to enhance the durability, stability, and reusability of various enzymes, even in harsh reaction conditions, such as extreme pH, temperature, and salts [13]. Polysaccharides, including collagen, chitin, and cellulose, have been identified as having the potential for protein immobilization [14]. For instance, cellulose was positively charged with polyethyleneimine (PEI), an effective ionic polymer for the development of immobilized enzyme biocatalysts [15]. The cellulose-PEI composite presents notable benefits for enzyme immobilization, owing to its hydrophilic nature, which is attributed to the presence of hydroxyl and imino groups. This property shields the enzyme molecule from unfolding during immobilization, as reported by Martino’s group [16].

Dextran and cellulose are classified as glucans with distinct glucosidic bonds. The aim of this study was to establish a technique for generating insoluble dextran, given that low-molecular-weight dextran has the capacity to form microspheres or gels that exhibit superior stability. Additionally, the structural properties of dextran TB-7 in different morphologies (S-dextran and IS-dextran) were analyzed. Finally, the potential for IS-dextran to be positively ionized with polyethyleneimine was explored, with the goal of utilizing IS-dextran-PEI as a promising support for enzyme immobilization.

## 2. Results and Discussion

### 2.1. Synthesis of Dextran by DSR-MΔ2-K654A

**Figure 1:**
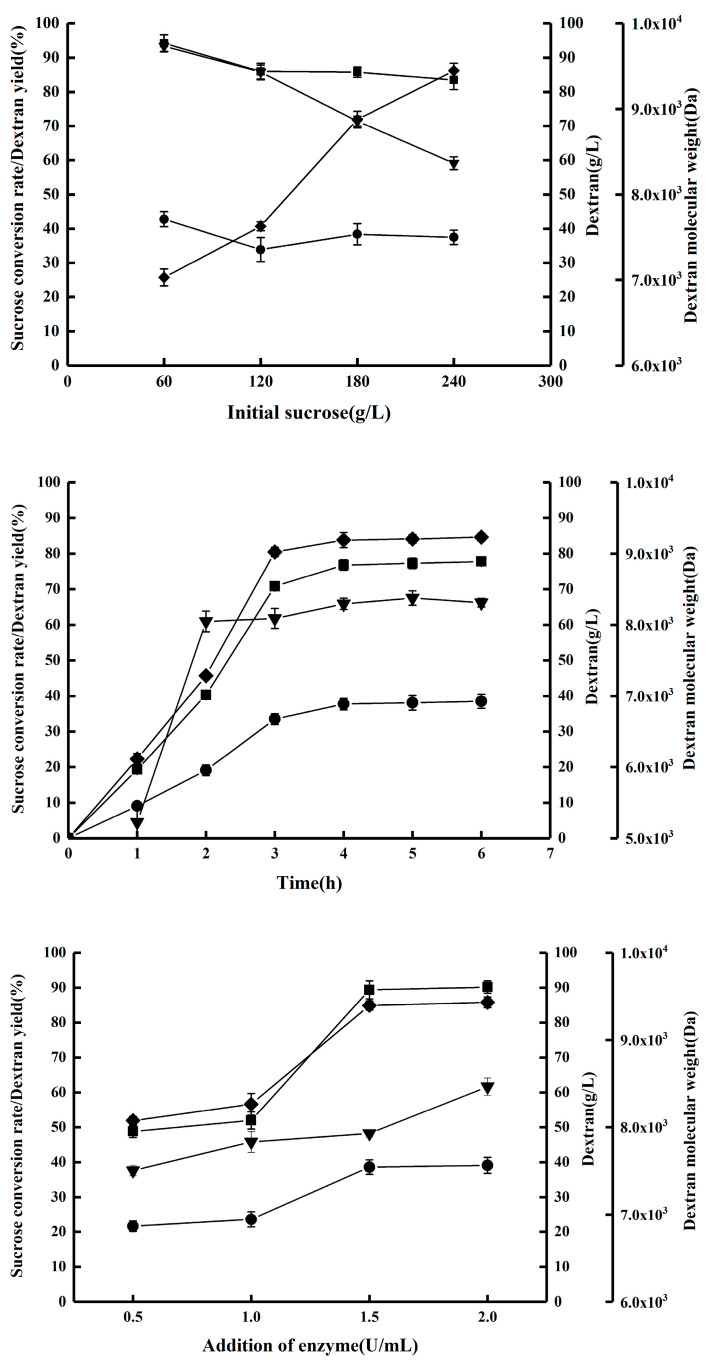
Effects of initial substrate, addition of enzyme, and the reaction time on sucrose conversion rate (●), dextran yield (■), dextran concentration (◆), and dextran molecular weight (▼).

In a previous study, we utilized a dextransucrase encoded by dsr-MΔ2-K654A (sequence provided in Supplementary Materials) to catalyze the conversion of sucrose to dextran with a molecular weight range of 7000~9000 Da [17]. In this current investigation, we examined the impact of initial sucrose concentration, enzyme quantity, and reaction duration on dextran molecular weight. Our findings, as depicted in Figure 1, indicate that dextran molecular weight increased as the initial sucrose concentration decreased and enzyme quantity increased. Furthermore, the growth pattern of dextran followed a linear model for the initial 3 h period, after which it stabilized at a consistent level. The production yield of dextran was predominantly influenced by the duration of the reaction and the quantity of enzyme added. Consequently, in order to achieve a high yield of dextran with a relatively low molecular weight, the enzymatic reactions were conducted using 240 g/L of sucrose for a duration of 4 h, with the addition of two units of enzyme per liter of reaction mixture.

### 2.2. Preparation and Storage of Purified Dextran Solution

**Figure 2:**
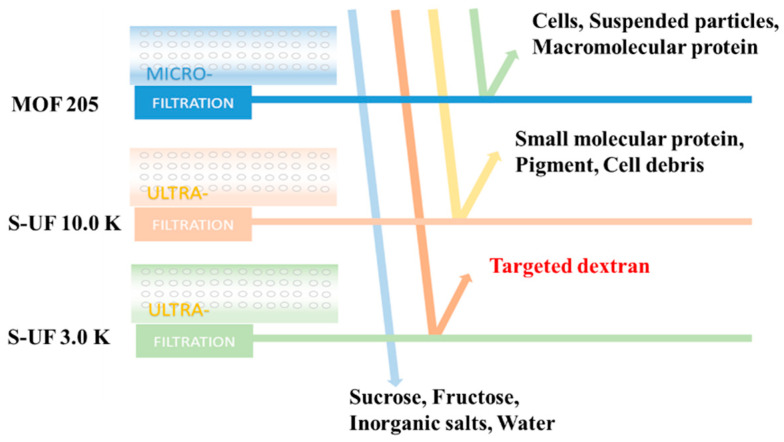
Purification process for dextran with molecular weight between 6000 and 8000 Da.

The enzymatic reactions were conducted using optimized conditions, and the purification process was executed through membrane-separation technology. Figure 2 illustrates the sequential passage of the enzymatic reaction mixture through microfiltration and ultrafiltration membranes, with the collected trapped fluid of the S-UF 3.0 K ultrafiltration membrane also depicted.

**Figure 3:**
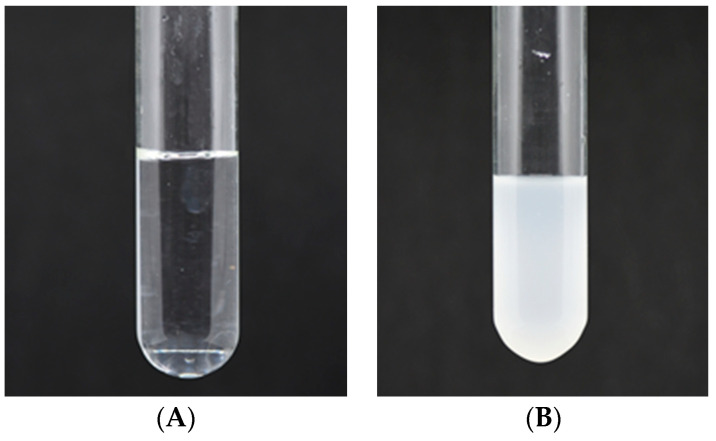
Dissolution characteristic of dextran solution before storage (**A**) and after one week of storage (**B**).

Stenekes et al. have previously reported that dextran with a molecular weight of 6000 Da can be easily formulated into hydrogels and microspheres through crystallization [9]. Thus, the collected dextran solution was stored at 4 °C to investigate the possibility of precipitation. After one week, the solution exhibited turbidity, as depicted in Figure 3, in comparison to its pre-storage state. Therefore, it is imperative to establish an appropriate storage method for this dextran, particularly if it is intended for the production of medical products, such as the widely used anti-anemia drug iron-dextran, which necessitates high solubility [18].

Dextran that contains a high ratio of α-(1,6) bonds exhibits a propensity to dissolve in aqueous solutions. In this study, IS-dextran was synthesized, and two theoretical models were proposed to account for this behavior. The first model posits that intermolecular hydrogen bonding enhances the cohesion between dextran particles, resulting in precipitation. The second model suggests that the ordered long-chain structure of dextran is predisposed to form crystalline regions, which serve as nuclei for crystal growth [19].

### 2.3. Effects of Drying Rate on the Formation of Dextran Precipitates

Due to the instability of dextran solution during storage, it is advisable to prepare it in powder form. Hirata et al. have reported that the generation process of dextran precipitates involves an adsorption process at the air–liquid interface to formulate dextran nuclei, followed by an elongation process that leads to the precipitation of dextran [7,8]. Consequently, the formation of the cross-linking structure that results in precipitation requires sufficient time, and it is plausible that the rate of drying may impact the formation of dextran powder.

Then, two drying methods, namely spray drying and oven drying, were utilized to produce dextran powder. The resulting spray-dried dextran (S-dextran) was found to be water-soluble, while the oven-dried dextran (IS-dextran) was insoluble. Subsequently, S-dextran was dissolved to prepare dextran solutions of varying concentrations (90, 120, and 240 g/L) to examine the impact of concentration on storage. The results presented in Table 1 indicate that a concentration of 240 g/L yielded 28.71% IS-dextran, which is consistent with the hydrogen-bond crystallization theory. Specifically, a greater polymer/water ratio prompts chain association via hydrogen bonds, culminating in crystallization.

Moreover, this study explored the impact of drying rate and concentration on the morphology of dextran. The findings presented in Table 2 indicate that all dextran powder produced via spray drying could be fully redissolved in water, while dextran powder obtained through oven drying resulted in significant precipitates, with a yield exceeding 60%. This study offers a novel approach to enhance the formation of insoluble dextran by regulating the drying rate, which differs from the results reported by Stenekes et al. that suggested that 6000 Da dextran solutions below 300 g/L would not form precipitates [9].

### 2.4. Characterization of Insoluble Dextran Precipitates

In recent years, a significant portion of research has been dedicated to examining the underlying mechanisms involved in the formation of IS-dextran in order to prevent its occurrence. In this study, we conducted a thorough characterization of insoluble dextran precipitates and explored the potential application of IS-dextran as a dermal filler.

#### 2.4.1. Solubility of IS-Dextran in Different Solvents

The solubility of IS-dextran was initially assessed in various solvents, including distilled water, 0.5 M NaOH solution, 0.5 M NaOH and 1 M urea mixed solution, DMSO, and ethanol. The results presented in Table 3 indicate that IS-dextran is solely soluble in DMSO, indicating its high stability in alkaline and alcoholic environments. This observation suggests that the structure of IS-dextran differs from the crystallization that typically results from hydrogen bonding.

#### 2.4.2. Molecular Weights Analysis

S-dextran and IS-dextran were dissolved in DMSO and subjected to gel chromatography. The results indicated that the average molecular weight of IS-dextran was 6555 Da, which closely approximated that of S-dextran. Nevertheless, the distribution coefficient (PDI) for IS-dextran was found to be larger than that of S-dextran, as shown in Table 4.

#### 2.4.3. SEM Analysis

**Figure 4:**
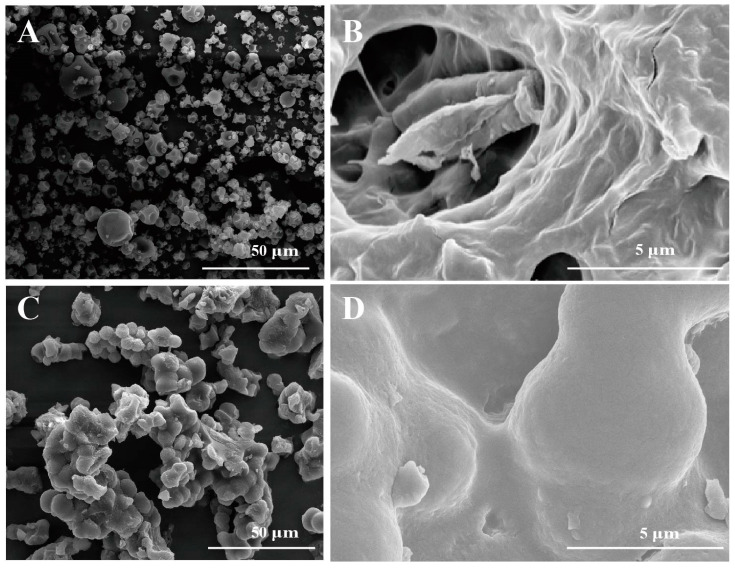
SEM of S-dextran and IS-dextran. ((**A**) Soluble dextran with a magnification of 2000; (**B**) Soluble dextran with a magnification of 10,000; (**C**) Insoluble dextran with a magnification of 2000; (**D**) Insoluble dextran with a magnification of 10,000).

The SEM results indicated that S-dextran exhibited a regular polyhedral shape and formed a hollow structure, as depicted in Figure 4A,B. Conversely, IS-dextran displayed an irregular and dense structure, as shown in Figure 4C,D. This characteristic may explain the water insolubility of IS-dextran, as the compact structure of IS-dextran impedes the entry of water molecules, in contrast to S-dextran.

#### 2.4.4. Spectroscopic Analysis

**Figure 5:**
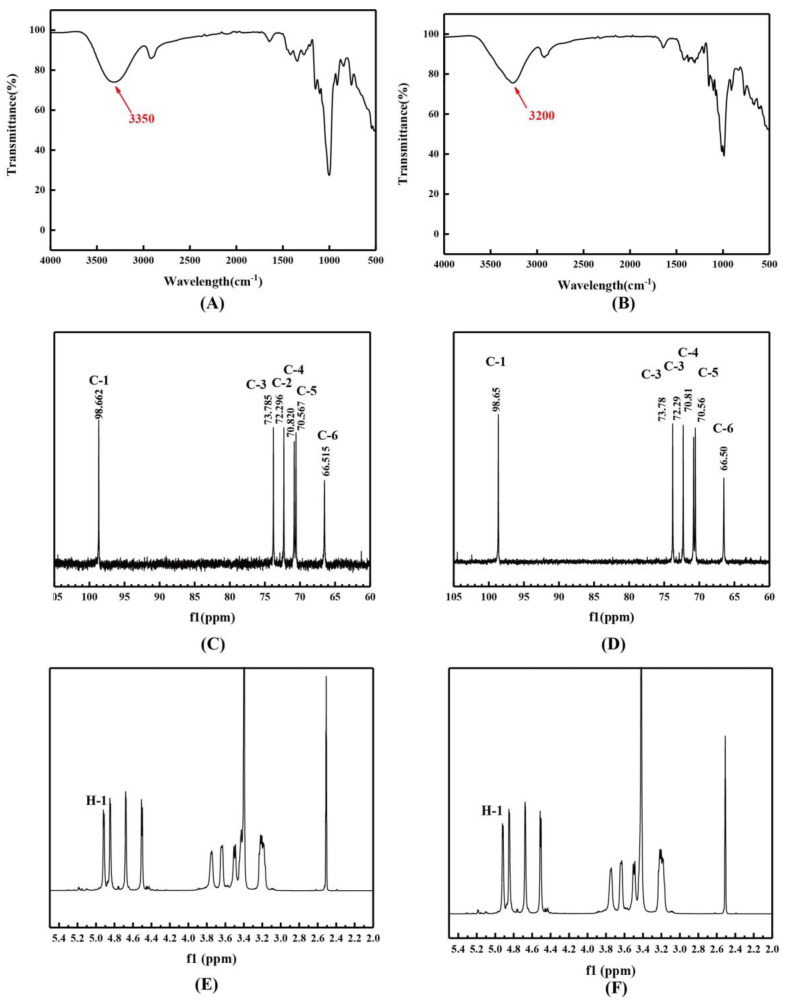
Infrared spectra of S-dextran (**A**) and IS-dextran (**B**), ^13^C NMR of S-dextran (**C**) and IS-dextran (**D**), and ^1^H NMR of S-dextran (**E**) and IS-dextran (**F**).

As shown in Figure 5A,B, it is evident that the O-H band for S-dextran was observed at 3350 cm^−1^, while IS-dextran exhibited a chemical shift to 3200 cm^−1^. Additionally, IS-dextran displayed sharper peaks compared to S-dextran, indicating restricted atom mobility and crystallinity. The absorption bands in the 1200–700 cm^−1^ region corresponded to C-O-C and C-O stretching, as well as C-C-H, C-O-H, and H-C-O deformation, which are characteristic regions of sugar rings and glycosidic bonds [20,21]. The identification of unique bands of S-dextran and IS-dextran at 1101, 915, and 762 cm^−1^ provided evidence that the glycosidic bond type was an α-(1,6) bond [22]. The absence of a characteristic peak of an α-(1,3) bond in the spectrum of IS-dextran indicated that its insoluble property was not a result of glycosidic bond transformation from an α-(1,6) to an α-(1,3) linkage. The NMR spectrum demonstrated that the peak positions of both soluble and insoluble dextran were virtually indistinguishable, suggesting that both were α-glucans connected by α-(1,6) bonds (Figure 5C–F). This was corroborated by the absence of α-(1,3) bonds in IS-dextran.

#### 2.4.5. DSC and XRD Analysis

**Figure 6:**
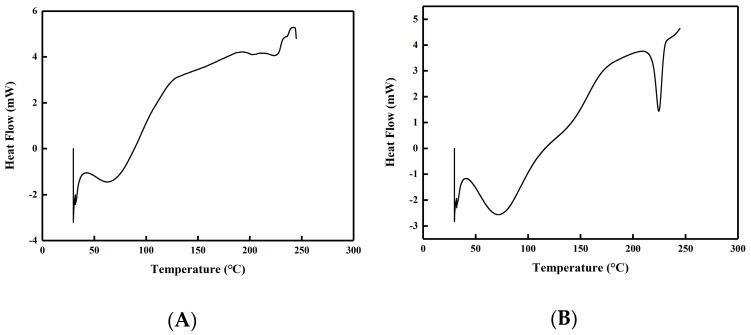
DSC thermograms of S-dextran (**A**) and IS-dextran (**B**).

**Figure 7:**
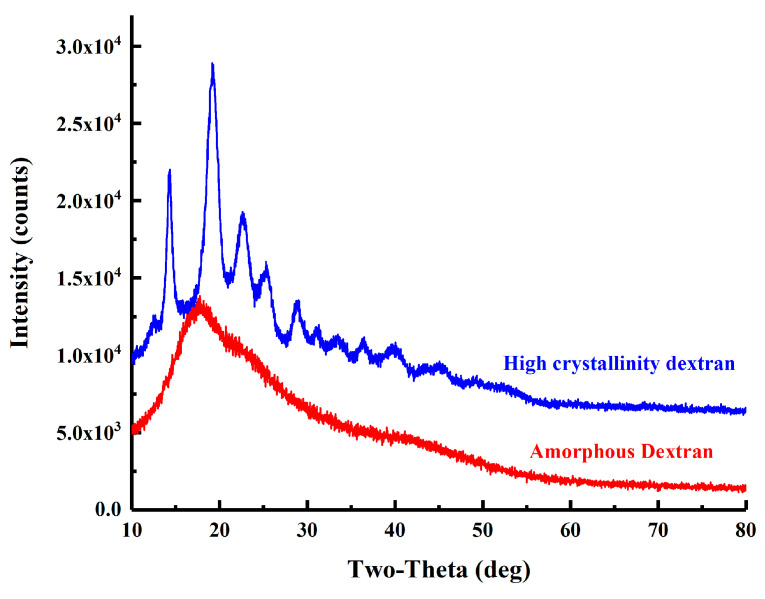
XRD spectrum of S-dextran and IS-dextran.

As shown in Figure 6, both S-dextran and IS-dextran exhibited a peak within the temperature range of 30–150 °C, which can be attributed to the evaporation of residual water. Additionally, an evident peak was observed in the 210–240 °C range for IS-dextran, indicating that it possesses at least partial crystallinity. The crystalline nature of IS-dextran was further confirmed by the XRD spectrum presented in Figure 7. Specifically, the XRD spectrum of S-dextran displayed a sole dispersion peak and no prominent diffraction peak, indicating an amorphous structure lacking crystal lattice. In contrast, IS-dextran had multiple diffraction peaks at 14.36°, 19.3°, 22.76°, 25.5°, and 29°, which is similar to cellulose I and II, and the calculated crystallinity reached 93.62% [23,24].

Microcrystalline cellulose is composed of cellulose crystals that possess properties such as reproducibility, non-toxicity, a highly specific surface area, and biodegradability, which confer a multitude of advantages, including drug release and capacity capabilities [25]. The IS-dextran in this study shares several analogous characteristics with microcrystalline cellulose, as both belong to the glucan family and exhibit high crystallinity while maintaining stability in alkaline and alcoholic environments.

#### 2.4.6. Zeta Potential Analysis

**Figure 8:**
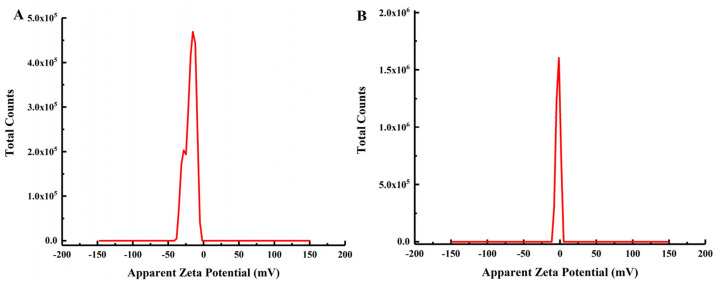
Zeta potential of S-dextran and IS-dextran ((**A**) S-dextran; (**B**) IS-dextran).

The zeta potentials of S-dextran and IS-dextran were analyzed, and the results indicated that at a neutral pH of 7, S-dextran exhibited a zeta potential of −18.5 mV, while IS-dextran exhibited a potential of −2.7 mV (Figure 8). These findings suggest that the dispersibility of IS-dextran in water is comparatively diminished, thereby increasing the likelihood of aggregation, as evidenced by its lower zeta potential, which is consistent with previous reports [26].

### 2.5. Preparation of Positively Charged IS-Dextran with PEI

Through the process of slow evaporation, a high yield of crystalline IS-dextran exceeding 60% was achieved (Table 2). IS-dextran, as a “low-active” polysaccharide, can be utilized as a carrier primarily after derivatization with amphiphilic groups. Due to the comparatively low negative zeta potential of −2.7 mV of IS-dextran, it is crucial to develop a proficient method for generating positively charged IS-dextran as a support for immobilizing enzymes. PEI has been proven to be a highly advantageous ionic polymer in the construction of immobilized enzyme biocatalysts [15].

As shown in Table 5, IS-dextran was successfully rendered positively charged with PEI, and the incorporation of epichlorohydrin resulted in all zeta potentials of IS-dextran-PEIs exceeding +30 mV. Zeta potential plays a crucial role in determining the physiological stability of cationic polymers. For instance, cationic dextrans such as CDI-dextran (C-Dex) and EDTA-dextran (EDTA-Dex), which possess a zeta potential of approximately 30–35 mV, have demonstrated the ability to disrupt biofilms and effectively control wound infections [27]. However, the zeta potential of dextran PEI imidazole (DPI) was found to be lower, ranging from 16–22 mV, which is below the values observed in this study [28]. Furthermore, small-branched PEI units (800/2000 Da) were conjugated to dextran (15,000/100,000–200,000 Da) to form dextran–polyethylenimine (Dex-PEI) conjugates, which showed zeta potentials lower than 25 mV [29].

Zaitsev et al. have reported that hydrolytic enzymes, such as lipases, can be efficiently immobilized with positively charged polysaccharides [30]. Also, Jin et al. have investigated the self-assembly behaviors of α-amylase and positively charged polysaccharides, resulting in the fabrication of α-amylase/polysaccharides complex coacervates. Remarkably, these coacervates retained 70% of enzyme activity even after exposure to pH 3.0 [31]. Consequently, IS-dextran-PEIs emerge as promising support for immobilizing enzymes with negative potential. Furthermore, given that the cell membrane surface is predominantly negatively charged, IS-dextran-PEIs can also potentially immobilize cells.

## 3. Materials and Methods

### 3.1. Strains, Plasmids, Media, and Conditions

The plasmid pET-28a-*dsr*-MΔ2-K654A was constructed previously [17]. *E. coli* BL21 (DE3) was used as the host for the expression of enzyme DSR-MΔ2-K654A.

*E. coli* strains were cultured in Luria–Bertani (LB) medium (10 g/L of tryptone, 5 g/L of yeast extract, and 10 g/L of NaCl) with 0.1 g/L of kanamycin at 37 °C for growth, with shaking at 200 r/min. When optical density at 600 nm (OD_600_) reached 0.6–0.8, 0.05 mM of IPTG was added for 24 h enzyme expression at 25 °C. Then, cells were harvested by centrifugation, resuspended in buffer (20 mM phosphate sodium buffer, pH 7.0), and disrupted by sonication. Recombinant dextransucrase was recovered at 12,000 r/min for 10 min and purified by ammonium sulfate precipitation and Sepharose 6B gel filtration chromatography [32].

### 3.2. Dextransucrase Activity Assay and Reaction Condition

The activity of dextransucrase was determined by the dinitrosalicylic acid method, and one unit was defined as the amount of enzyme that catalyzed the formation of 1 μmol of fructose per minute from 584 mM of sucrose in 50 mM sodium acetate buffer at pH 5.8 [33].

Optimized enzymatic reactions were conducted utilizing 50 mM sodium acetate buffer at pH 5.8 under 30 °C, with varying amounts of initial sucrose and enzyme.

### 3.3. Preparation of Purified Dextran Samples

The reactions were terminated after 30 min of incubation at 100 °C, and dextran purification was achieved through membrane-separation technology. Figure 2 illustrates the sequential passage of the raw dextran solution through a microfiltration membrane (MOF 205) and two ultrafiltration membranes (S-UF 10.0 K, S-UF 3.0 K) with molecular weight cut-offs of 10,000 Da and 3000 Da, respectively. The experimental conditions involved maintaining a temperature below 40 °C and an operating pressure below 0.3 MPa. The target product solution was confined within the trapped fluid of the S-UF 3.0 K ultrafiltration membrane. Subsequently, the purified dextran solution underwent drying via two methods: spray drying and oven drying. The former method, which is a rapid evaporation process, yielded dextran powder in a matter of minutes, while the latter, which is a slow evaporation process, required approximately two days with the dextran solution being maintained at around 80 °C. The resulting dextran powders were assigned corresponding names: S-dextran and IS-dextran.

### 3.4. Solubility of SI-Dextran in Different Solvents

Five serum bottles were prepared, each containing 0.5 g of dextran precipitate. Subsequently, 50 mL of distilled water, 0.5 M NaOH solution, 0.5 M NaOH and 1 M urea mixed solution, DMSO, and ethanol were added sequentially to each bottle. The serum bottle containing distilled water, NaOH solution, NaOH-urea mixed solution, and DMSO was subjected to heating at 80 °C for 15 min, while the serum bottle containing ethanol was heated at 40 °C for 15 min.

### 3.5. Gel Chromatography Technology

Gel permeation chromatography was utilized to determine the molecular weight of soluble dextran powder. The mobile phase, consisting of reverse osmosis water filtered at 0.45 μm and degassed, was employed at a flow rate of 0.5 mL/min. Prior to analysis, both soluble and insoluble dextran were dissolved in DMSO by heating at 70 °C for 15 min, followed by filtration (0.45 μm). The mobile phase for this step was degassed DMSO, also at a flow rate of 0.5 mL/min. The column was calibrated using a set of standards with a narrow molecular weight distribution.

### 3.6. Infrared Spectrum

Infrared spectroscopy analysis was conducted using a Nicolet IS 10 instrument. The S-dextran and IS-dextran samples were blended with dried KBr powder in a 1:100 ratio and subsequently compressed into 1 mm pellets prior to scanning. The samples were scanned over a range of 400 cm^−1^ to 4000 cm^−1^ with a resolution of 4 cm^−1^.

### 3.7. SEM, Scanning Electron Microscope

The soluble powder and sediment were characterized using a scanning electron microscope (Japanese position emission scanning electron microscope SU8010, Hitachi, Tokyo, Japan) at magnifications ranging from 400 to 2000. Particle size was determined using an image analysis program. 

### 3.8. Zeta Potential

The soluble and insoluble dextran samples were analyzed using a zeta potential analyzer (Malvern Zetasizer Nano ZS-90, Malvern Instruments, Worcestershire, UK) with water as the dispersant. The zeta potential was estimated using the Smoluchowski equation.

### 3.9. Differential Scanning Calorimetry

A differential scanning calorimeter (DSC Q2000 V24.11 Build 124, NAICHI, Shanghai, China) was used to analyze soluble dextran powder and insoluble precipitates. To ensure greater accuracy of data, the modulation mode was employed. The sample was subjected to heating from 30 °C to 250 °C at a rate of 3 °C/min, with 4 modulations per minute and a modulation amplitude of 0.5 °C, while being maintained in a nitrogen atmosphere.

### 3.10. X-ray Diffraction

The soluble dextran powder obtained via spray drying and the insoluble dextran powder obtained through gradual dehydration, which underwent grinding in a quartz mortar and subsequent sieving, were subjected to XRD analysis (Smartlab 9 kw, Rigaku, Tokyo, Japan). The diffraction data were collected from 10 to 80° at a scanning speed of 10°/min.

### 3.11. Coating IS-Dextran with PEI

To synthesize IS-dextran-PEIs, 1 g of dextran and 1 g of PEI with varying molecular weights (600 Da, 1800 Da, and 10,000 Da) were individually dissolved in 50 mL of deionized water, mixed, and stirred thoroughly to obtain a homogeneous solution. The pH of the mixture was adjusted to 7 using NaOH solution. Five-milliliter epichlorohydrin was then selectively added dropwise to the reaction solution, followed by gentle stirring for 24 h. The pH of the reaction solution remained constant throughout the reaction. Upon completion of the reaction, the resulting IS-dextran-PEIs were collected by centrifugation to analyze the zeta potential.

## 4. Conclusions

The control of polymorph formation in polymers is a matter of great significance due to the diverse physical properties exhibited by distinct polymorphs, such as water solubility, stability, and bio-availability. This study describes an efficient enzymatic synthesis and a membrane-based separation method of soluble dextran (6702 Da), aligning with the principles of sustainable manufacturing. Also, this study has successfully developed a method for producing insoluble dextran, which generally possesses high water insolubility, through the process of slow evaporation, with a yield exceeding 60%. The resulting IS-dextran has an average molecular weight of 6555 Da and a calculated crystallinity of 93.62%. SEM results revealed that IS-dextran possesses an atypical and compact configuration, which explains its lack of solubility in water. 

Additionally, the introduction of a positive-charge modification to IS-dextran, in conjunction with the integration of epichlorohydrin, resulted in all zeta potentials of IS-dextran-PEIs surpassing 30 mV, thereby establishing it as a promising support for enzyme immobilization. Despite the impressive physiochemical properties demonstrated by IS-dextran-PEIs, the selection of raw materials and preparation methods, as well as the structure of the raw materials, can potentially impact the characteristics of cationic dextrans. Therefore, it is necessary to conduct comprehensive investigations in these areas. In order to utilize cationic polymers effectively, further research can be conducted using insoluble dextrans as drug carriers and nano-materials. Additionally, it is important to consider the potential limitation of storing purified dextran solutions, taking into account the underlying mechanism of insolubilization. Consequently, we have identified a novel variety of environmentally sustainable bio-based materials, which warrants further exploration in various fields, including medicine, food, and other industries.

## Figures and Tables

**Table 1:** Yield of IS-dextran with different initial concentrations of dextran solution after one week of storage at 4 °C.

Initial Concentration of Dextran Solution (g/L)	Yield of IS-Dextran (%)
90	0.51 ± 0.05
120	1.30 ± 0.11
240	28.71 ± 5.38

**Table 2:** Yield of IS-dextran by spray drying and oven drying with different concentrations of dextran solution.

Initial Concentration of Dextran (g/L)	Drying Method	Yield of IS-Dextran (%)
90	Spray drying	N.D.
90	Oven drying	59.1 ± 5.3
120	Spray drying	N.D.
120	Oven drying	65.8 ± 4.9
240	Spray drying	N.D.
240	Oven drying	67.4 ± 4.1

N.D. means not detected, that is, no dextran precipitates formed by spray drying.

**Table 3:** Solubility of IS-dextran in different solvents.

Solvent	Condition	Solubility
Distilled water	80 °C,15 min	Insoluble
0.5 M NaOH	80 °C, 15 min	Insoluble
0.5 M NaOH + 1 M Urea	80 °C, 15 min	Insoluble
DMSO	80 °C, 15 min	Dissolved completely
Ethanol	40 °C, 15 min	Insoluble

**Table 4:** Analysis of molecular weights and polydispersity of S-dextran and IS-dextran.

	Mw (Da)	PDI
S-dextran	6702 ± 274	1.64 ± 0.09
IS-dextran	6555 ± 155	1.98 ± 0.06

**Table 5:** Preparation of positively charged IS-dextran with PEI.

	Condition	PDI
IS-dextran-PEI600	PEI600	20.9 ± 2.1
IS-dextran-PEI1800	PEI1800	34.4 ± 3.9
IS-dextran-PEI10,000	PEI10,000	37.7 ± 2.4
IS-dextran-PEI600	PEI600 + Epichlorohydrin	34.7 ± 3.5
IS-dextran-PEI1800	PEI1800 + Epichlorohydrin	39.6 ± 3.1
IS-dextran-PEI10,000	PEI10,000 + Epichlorohydrin	35.3 ± 3.3

## Data Availability

Not applicable.

## Supplementary Materials

The following supporting information can be downloaded at https://www.mdpi.com/article/10.3390/molecules28207210/s1: Sequence S1: Gene sequence encoding enzyme DSR-mTB7; Figure S1: Left is the infrared spectrum of soluble dextran (Red) and insoluble dextran (Blue); Right is the NMR spectrum: A is ^1^H-NMR of soluble dextran in DMSO, B is ^13^C-NMR of soluble dextran in DMSO, C is ^1^H-NMR of insoluble dextran in DMSO, and D is ^13^C-NMR of insoluble dextran in DMSO.
