# Supplementary material for: Preparation and Characterization of a Novel Morphosis of Dextran and Its Derivatization with Polyethyleneimine
Source: Molecules. 2023 Oct 21;28(20):7210. doi: 10.3390/molecules28207210 (PMC10609354; doi:10.3390/molecules28207210)
Supplement: Supplementary file 1 [file molecules-28-07210-s001.zip › molecules-2615819-supplementary.pdf]

Sequence S1: Gene sequence encoding enzyme DSR-mTB7.

atggcaagctttgaaaaagcccctgattcggttcccgaaactataactggtgggcgttatagtttgaaagatggctattacgtttatctggacaag
caagggaagcaagttgttggccctaaaaacattgataatcatctccaatattttgatgaaacaacgggcaaacaagttaaaggagattttcgtag
tgttaacggtaaacgtatttatttcaatgctaacttaggttacgcagatgattatacgaccgatgttgctggtaagttagttggttacgattctaatggt
aatcaggtaaaagcaggctatgttactaacagtcagggaaaaacatactactttaataaccaaggcgaggcgattattggtttaaaaactgataa
caacaagacgcaatattttggtcccgatggtgcgcaagttaaaggtgcttttcaacaagttaatggaaaaaacatttattttgatgctcaaactgga
tacgctagacaaaatgtaggatttttggatggtacagcaaaagggtttgatgagcaaggaaatcagattaaaagtggtatagccactgatttgtc
aggtaatgtttactattttgatgctagtggaaagatgttaacaggcgttcaaaatattgatggcaagaaatattactttgatgaacagggacatcgt
agaagaaattatgctggtgtatttaataatgaatttatttactttggattagatggcgttgggcaaagtgccattgaataccagtttgagaaaggatt
aacttcacaaaacagtgttgctacaagtcataatgctgcaaagtcttatgataccaaaagttttactaacgtggatggtttttaactgctaattcatg
gtatcgacctaccgacattttaagaaatggcacaaagtgggagccttcgacagaaactgattttaggccactgctcatgacttggtggcctgata
aagaagtacaggcgaattatttgaactatatgtctgcgctaggactgggtgatcaaaaaatatatacgggggcctcgagtcaattagacttaaat
aatgctgctttgattgttcaagaagccattgaaaaaaagattagtcttgaaaaaagcacaaaatggttagacgattccattaaaagttttattaaaa
gcaaacgcaaagatattcagggaaacttggtagacaccaacccagggtggacgattgatagtgaaacaggctctactaaccatttgcaaaatg
gggcgtttatctttacaaatagtcccttagttcctgaagcaaatgcagcagaaggtaaccgattaattaacaggacacctagtcaacagacggg
aaatcatatatcatatgcaagccaaccgtacagcggagacgattggggatatgaactattattaggcaatgatgtcgataattctaatcctatcgt
acaagctgaacaactaaactggatacattatttgatgaattttgggacgataacggcgcctcaggatccagacgcacatttagctaattttgatag
cattcgaattgacgcagtagataatgttgatgctgacttattacagattgccggcgattattttaaagctgcttatcaggtaggagaaaacgataaa
aatgcgaatcaacacattcacattttagaagattggtctcctaatgacgtttggtataaccaacaagttaatggtaatagccaattaactatggatg
ccacgatgcaaaaccaattgttagcatcattaacgagacccattaccagtagagattctatgaagagttttactaaagacgctctgctagttcatc
gaactgctgataattcttacaatcaggccgtacccaattacagctttattcgagctcatgatagtgaggttcagacaataattgccaaaattatttct
gataagcatcctgatttatatcccactgttgataaggctttactggctaaggatagtgccctctacgacgaagcttttacagagtataatgctgaca
tgcaaaagatttcttcacaaaagcagtatacgcataataatatgcccagtgcttatgcaattttgttaactaataaagatactgtgccaagagtctat
tatggtgatttatttacagataatggtgagtatatggctaataagacgccttattacgatgccatcacgagtttgcttaccgcacgtaccaaatttgt
atcaggtggacaatcgctttccgtagataagaatgatgtgttaactagtgtcagatacggaaaaggtgccttgtctgcaacggataacggtagtt
ctgacacacgtaatcaaggcattggtgttattgtcagtaataatcctaatttggatttaaataacgataaagtgactttgagcatggggattagtcat
gcacatcaagcataccggcctttattattaactaacagtcagggaatagtggcatatgcaacagacagcgaagtaccacagaatctttataaaa
caactaatgataaaggtgaattgacgtttgatgcatcagagataaaaggttatgatactgttcagacatctggttacttagctgtatgggtaccggt
aggcgcttctgatgaacaagatgctagaaccatagccagtactgaaaaaaataatggtaattctgtttatcattctaatgctgcattggattctcaa
cttatctatgaaggattttctaattttcaaactgtcccatccaaaaatgcttcggcagatgaatatgccaacgttattattgcaaaacatgctgcaga
ctttaataaatggggtgttacaagtttccaaatggcgccacaatatcgctcaagtactgacggctcatttttggacgcagtagatactgttcaaaat
ggttatgcgtttactgaccgctatgatttaggatttaatgcagcagatggttctaaaaatcctacaaaatacggaacagatgaggacttaagaaat
gccattaaatctcttcatgctcagaaaacatatgatggttcaagtatacaagtaatggctgattttgttcctgatcaactttataatatgcctttggaac
aagcagtttctgtcattagaacagataaatacggtgttaattcagaaaatccagatattcaaaatattatttatgctgcgaatataaaaagcagtgg
cactgattatcaatcaatatatggtggaaagtatttagctgagctacaaaaaaatccattatttaaatcattgtttgatcgaatacaaatttctactaaa
aaaacgattgatccgaatactagaattacacaatggtccgctaaatattttaatggttctaacattcagggaaaaggaatcaactacgtcttaaaa
gactgggcatctaataagtattttaatgtttctagcaacgatgatatgtattctcgtctgcccaagcagcttatgaatcaagagtcaaatactgggtt
tattgttgatgatatcggtgtaaaatactattcaattagtggttatcaagcaaaaaacacatttgttgaggatggtaacggagagtggtactactttg
ataatgatgggtatatggtgaaatccactgaagagagtggccctttaagaactgtgaacgcgtcttctaagaaatattatattctgccaaatggtgt
tgaaataaggaacagtttcggtcaagatattcagggaaatacctactatttcgatgcacgtggtgaaatggtaaccagtcagtatatttcagatga
tacacaaaatatttactattttaataacgatggtacgatggcaaaataa

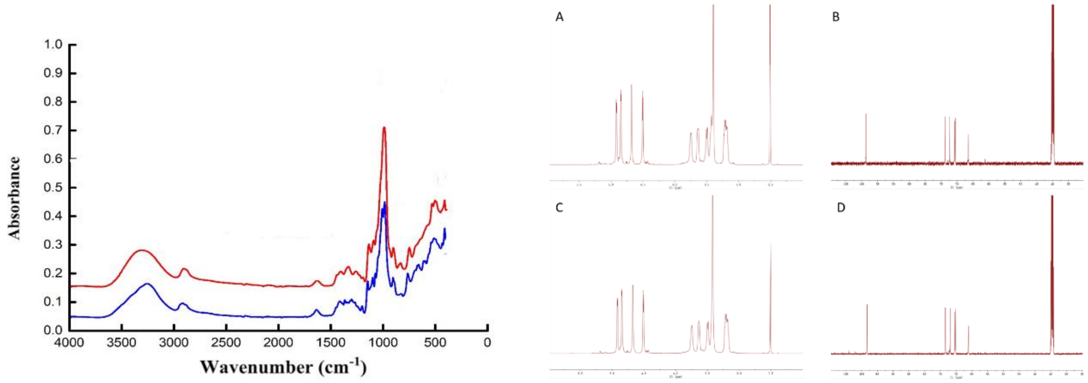

Figure S1. Left is infrared spectra of soluble dextran (Red) and insoluble dextran (Blue); Right is the NMR spectrum: A is ^{1}H-NMR of soluble dextran in DMSO, B is ^{13}C-NMR of soluble dextran in DMSO, C is ^{1}H-NMR of insoluble dextran in DMSO, and D is ^{13}C-NMR of insoluble dextran in DMSO.
